# Gut Microbiota, Metabolic Markers, and Systemic Inflammation in Young Women with Self-Reported Rosacea: An Exploratory Cross-Sectional Study

**DOI:** 10.3390/jcm15114130

**Published:** 2026-05-27

**Authors:** Paola Pavačić, Emanuela Krpan, Karlo Zeman, Romia Gregorović, Sara Kralj, Željana Bolanča, Valentina Borko, Helena Čičak, Ana-Marija Liberati Pršo, Olga Gornik, Andrija Karačić

**Affiliations:** 1 The Gut Microbiome Center, 10000 Zagreb, Croatia; 2 Poliklinika Bolanča, 10110 Zagreb, Croatia; 3 Department of Biochemistry and Molecular Biology, Faculty of Pharmacy and Biochemistry, University of Zagreb, 10000 Zagreb, Croatia; 4 Department of Medical Laboratory Diagnostics, Sveti Duh University Hospital, 10000 Zagreb, Croatia; 5 Department of Internal Medicine, Sveti Duh University Hospital, 10000 Zagreb, Croatia

**Keywords:** rosacea, gut microbiota, gut–skin axis, insulin resistance, metabolic health, obesity, *Bifidobacterium*, *Roseburia*, sleep quality, exploratory analysis

## Abstract

**Background:** Rosacea is a chronic inflammatory dermatosis with emerging links to the gut–skin axis, yet integrative data connecting fecal microbiota with metabolic and immune parameters remain scarce. **Methods:** Within a cohort of Croatian women (*N* = 300, aged 30–35), participants with self-reported physician-diagnosed rosacea (*n* = 19) were compared to controls (*n* = 281). Assessments included validated questionnaires, anthropometrics, fasting blood parameters, and fecal 16S rRNA gene sequencing (QIIME2). Taxonomic profiling at four levels (899 features) used Mann–Whitney U tests with per-level Benjamini–Hochberg FDR correction. This study follows STROBE and STORMS guidelines. **Results:** On the host side, rosacea was nominally associated with higher BMI (*p* = 0.037), poorer sleep quality (*p* = 0.038), elevated monocytes (*p* = 0.031), and higher HOMA-B (*p* = 0.013); these comparisons were not corrected for multiple testing. Fecal 16S rRNA analysis (rosacea *n* = 18, controls *n* = 265) identified 15 nominally significant genera, including depleted *Bifidobacterium* (*p* = 0.007), *Roseburia* (*p* = 0.033), and enriched *Anaerostignum* (*p* < 0.001). However, no taxon survived FDR correction at any taxonomic level (lowest q = 0.165), and the total number of nominally significant features (48/899, 5.3%) did not exceed chance expectation (binomial *p* = 0.340). All key taxa remained nominally significant after BMI adjustment. An exploratory Random Forest classifier (five genera + HOMA-B + cortisol) achieved LOOCV AUC = 0.785, but feature selection on the training data limits interpretation. **Conclusions:** In this narrowly age-defined female cohort, host-side metabolic and immune differences reached nominal significance without multiple-testing correction. Fecal microbiota differences were exclusively exploratory: no taxon survived FDR correction, and the overall signal did not exceed chance expectation. The pattern of findings is compatible with—but does not constitute evidence for—a gut–skin axis contribution to rosacea. Confirmation in adequately powered cohorts with clinical rosacea verification, comprehensive confounder capture, and pre-registered analyses is required before any mechanistic or clinical conclusions can be drawn.

## 1. Introduction

Rosacea is a chronic inflammatory dermatosis of the central face, characterized by persistent erythema, telangiectasia, papulopustular lesions, and phymatous changes, with an estimated global prevalence of 5.1% [1]. The condition predominantly affects women aged 20–50 years and substantially impairs quality of life through visible facial involvement and psychological distress [2]. Although rosacea classification has recently transitioned to a phenotype-driven approach [3], its pathophysiology remains incompletely understood. Current models implicate a complex interplay of innate immune dysregulation—with cathelicidin and kallikrein-5 overexpression—neurovascular hyperreactivity, genetic predisposition, and environmental triggers including chronic psychological stress, dietary factors, and sun exposure [4,5,6,7]. A recent consensus review identified persistent diagnostic and therapeutic gaps, underscoring the need for phenotype-directed management strategies and novel therapeutic targets [8]. In parallel, the role of the gut microbiome in modulating cutaneous inflammation has been increasingly recognized in the dermatology literature, with emerging evidence supporting microbial modulation as a complementary avenue warranting further investigation [9,10].

The concept of the gut–skin axis proposes that intestinal dysbiosis and increased gut permeability can modulate systemic immune responses and influence skin inflammation through metabolic, immunological, and neuroendocrine pathways [11,12]. As early as 1930, Stokes and Pillsbury proposed a gastrointestinal mechanism underlying emotional and nervous effects on the skin, anticipating the modern gut–brain–skin axis framework [13]. In rosacea, clinical evidence supporting this axis includes the observation by Parodi et al. that patients were 13-fold more likely to harbor small intestinal bacterial overgrowth (SIBO) than controls, and that SIBO eradication with rifaximin significantly improved cutaneous lesions [14]. Population-based cohort studies have consistently demonstrated that rosacea co-occurs at increased frequency with gastrointestinal conditions, including irritable bowel syndrome (IBS), gastroesophageal reflux disease (GERD), celiac disease, Crohn’s disease, and ulcerative colitis [15,16]. A recent narrative review reinforced that the rosacea–IBS association is likely mediated by shared genetic, microbial, and immune-related factors [17], and a large retrospective cohort study further reported an elevated risk of functional bowel disorders in rosacea patients, reinforcing the bidirectional nature of this relationship [18].

Despite growing clinical interest, fecal microbiota profiling studies in rosacea remain remarkably scarce. To date, nine case–control or interventional studies have examined the gut microbiota in rosacea—all with small sample sizes (*n* = 8–60 rosacea patients) and partly contradictory results. The earliest 16S rRNA studies in East Asian cohorts yielded discordant genus-level findings: Nam et al. found no diversity differences in 12 Korean women but identified decreased *Methanobrevibacter*, *Slackia*, and *Desulfovibrio* alongside increased *Acidaminococcus* and *Megasphaera* [19]. Chen et al. observed reduced richness in 11 Taiwanese patients alongside a strikingly different taxonomic profile—enrichment of *Bifidobacterium* and *Ruminococcus*, depletion of *Lactobacillus* and *Roseburia*, and, notably contradicting Nam et al., depletion rather than enrichment of *Megasphaera* [20]. Moreno-Arrones et al. provided a third perspective from a Spanish pilot study of 15 patients that confirmed beta diversity shifts but identified taxa with limited overlap [21]. Yilmaz et al. contributed early European data from a Turkish cohort, providing evidence that compositional differences may exist independent of East Asian ethnicity, although the study was limited in sample size and metabolic phenotyping [22]. The largest European cohort to date was reported by Guertler et al., who confirmed beta diversity differences but again identified taxa with limited overlap with Asian findings [23]. A systematic review by Sánchez-Pellicer et al. synthesized the available evidence, concluding that while a general pattern of dysbiosis may be emerging, no reproducible single-taxon signature has been established across studies, and emphasized the urgent need for larger cohorts, standardized methodologies, and integration of microbiota data with host metabolic profiling [24].

Recent studies have expanded the evidence with novel approaches. Joura et al. examined stool, blood, and skin microbiota simultaneously in 14 rosacea patients, representing the first multi-compartment approach, but found limited stool-level differences despite distinct skin microbiota profiles [25]. Yu et al. conducted a randomized controlled trial demonstrating that combined probiotic and doxycycline therapy improved skin symptoms while modulating gut microbiota composition in 60 Chinese rosacea patients, providing the first interventional evidence for gut–skin axis modulation in this condition [26]. Li et al. reported correlations between gut microbiota and serum metabolomics in neurogenic rosacea, identifying species-level taxa linked to neurotransmitter metabolism [27], and Zhao et al. characterized the gut microbiota in a Beijing cohort using whole-metagenome sequencing, identifying reduced *Roseburia* and altered KEGG pathways related to neuroinflammation [28]. A comprehensive review by Manfredini et al. further discussed the potential of probiotics and dietary interventions in rosacea, while acknowledging that the evidence remains preliminary [29]. Despite this growing body of literature, none of the published studies has simultaneously profiled the fecal microbiota alongside comprehensive metabolic and immune markers within a demographically homogeneous cohort.

Beyond microbiota, low-grade systemic inflammation in rosacea is supported by elevated laboratory inflammatory indices [30], and metabolic associations including insulin resistance have been documented in multiple cohorts. Sodagar et al. confirmed a meta-analytic association between metabolic syndrome and rosacea [31], and Akbaba et al. documented elevated HOMA-IR values, although with variable effect sizes [32]. Mendelian randomization studies suggest a causal trajectory from gut microbiota toward rosacea rather than the reverse [33], although such analyses are subject to well-known limitations including pleiotropy and instrument strength.

Against this background, the present study explored associations between gut microbiota composition, metabolic markers, immune parameters, and lifestyle factors in a narrowly age-defined cohort of young women (aged 30–35 years) with self-reported rosacea, integrating fecal 16S rRNA sequencing with comprehensive host phenotyping within a single demographically homogeneous sample. The main finding is a clear dissociation between the two data domains. On the host side, rosacea was nominally associated with higher BMI, poorer sleep quality, monocytosis, and elevated HOMA-B, with HOMA-B remaining nominally significant in a pre-specified three-group sensitivity analysis. On the microbiota side, although 15 genera reached nominal significance, no taxon survived FDR correction and the overall number of nominally significant features did not exceed chance expectation. The significance of this work therefore lies less in any single positive association than in demonstrating, within a deliberately homogeneous cohort, that previously reported rosacea–microbiota signals are not robust to multiple-testing correction, and in providing a transparently reported, adequately caveated reference dataset and analytical template for the design of future, adequately powered confirmatory studies.

## 2. Materials and Methods

### 2.1. Study Design and Population

This cross-sectional analysis was conducted within the project “The Glycome and Microbiome as Markers of Dietary Impact on the Health of Women of Reproductive Age.” The study enrolled adult women aged 30–35 years in Zagreb, Croatia. The protocol was approved by the Ethics Committee for Experimental Research of the Faculty of Pharmacy and Biochemistry, University of Zagreb, and all participants provided written informed consent. Exclusion criteria were pregnancy or lactation, acute infection (respiratory, gastrointestinal, or urinary tract), antibiotic administration within the preceding three months, and active oncological disease. A total of 300 women were enrolled, of whom 19 reported a prior physician diagnosis of rosacea; the remaining 281 served as the comparison group. Phylum-level nomenclature throughout follows SILVA v138.1, Max Planck Institute for Marine Microbiology, Bremen, Germany; recently proposed names (e.g., Actinomycetota for Actinobacteria) are noted where relevant.

Beyond the antibiotic exclusion criterion, current medication use, dietary supplement intake (including probiotics, prebiotics, vitamins, and minerals), use of proton-pump inhibitors, metformin, or hormonal contraceptives, and current dermatological treatment for rosacea were not systematically captured at enrollment in this cohort; the analytical and interpretive consequences of this gap are discussed in the Limitations (Section 4.4).

### 2.2. Case Ascertainment and Its Limitations

Rosacea status was determined by participant self-report of a previous physician diagnosis, collected through a structured questionnaire. No independent dermatological examination, photographic review, or phenotype subtyping was performed at the time of enrollment. We therefore use the term “self-reported physician diagnosed rosacea” deliberately and consistently, rather than treating the cases as clinically verified rosacea. This ascertainment method is a recognized source of misclassification bias that can operate in both directions: some women with genuine rosacea may have been classified into the comparison group (reducing sensitivity), and some women without current rosacea may have reported a past diagnosis that was incorrect, transient, or since revised (reducing specificity). Because rosacea is a phenotypically heterogeneous, fluctuating condition, self-report also cannot capture subtype (erythematotelangiectatic, papulopustular, phymatous, ocular) or current disease activity. We did not attempt to model or statistically “correct” this misclassification, as no validation subsample with gold-standard dermatological assessment was available; instead, we treat it as a fundamental constraint on internal validity. Consequently, all between-group comparisons in this study are framed as exploratory, the cases are described throughout as a self-reported group rather than a verified clinical population, and clinical verification is identified as the single most important requirement for any confirmatory study (Section 4.4).

### 2.3. Data Collection

Participants completed a secure online questionnaire covering demographics, self-reported physician-diagnosed conditions, smoking, and alcohol consumption. Dietary assessment used FAO-validated tools, including the Minimum Dietary Diversity for Women (MDD-W), the All-5 food group indicator, the Global Diet Recommendations (GDR) score, and the Mediterranean Diet Adherence Screener (MEDAS). Psychological well-being was assessed using the WHO-5 Well-Being Index, mental health quality of life using the MHQoL-7D and MHQoL-VAS, perceived stress using the Perceived Stress Scale (PSS), and sleep quality using the Sleep Quality Scale (SQS; range 0–84, with higher scores indicating poorer sleep quality). Personality traits were assessed using the Ten-Item Personality Inventory (TIPI).

### 2.4. Anthropometric and Laboratory Assessments

Standardized anthropometric measurements were performed at the Gut Microbiome Center, Zagreb, including body weight, height, and waist and hip circumferences, each measured in triplicate. Morning fasting venous blood was collected at University Hospital Sveti Duh for complete blood count with five-part differential, fasting insulin and glucose, cortisol, vitamin B12, and folic acid. Homeostatic Model Assessment indices were calculated: HOMA-B (β-cell function), HOMA-S (insulin sensitivity), and HOMA-IR (insulin resistance). Owing to budgetary constraints, extended panels—including serum calprotectin, IL-6, CRP, iron metabolism markers, and vitamin D—were available only for a subset of participants (*n* = 94–200 depending on the analyte); the exact denominator for each analyte is reported in the corresponding table footnote.

### 2.5. Fecal Sample Collection and 16S rRNA Gene Sequencing

**Figure 1:**
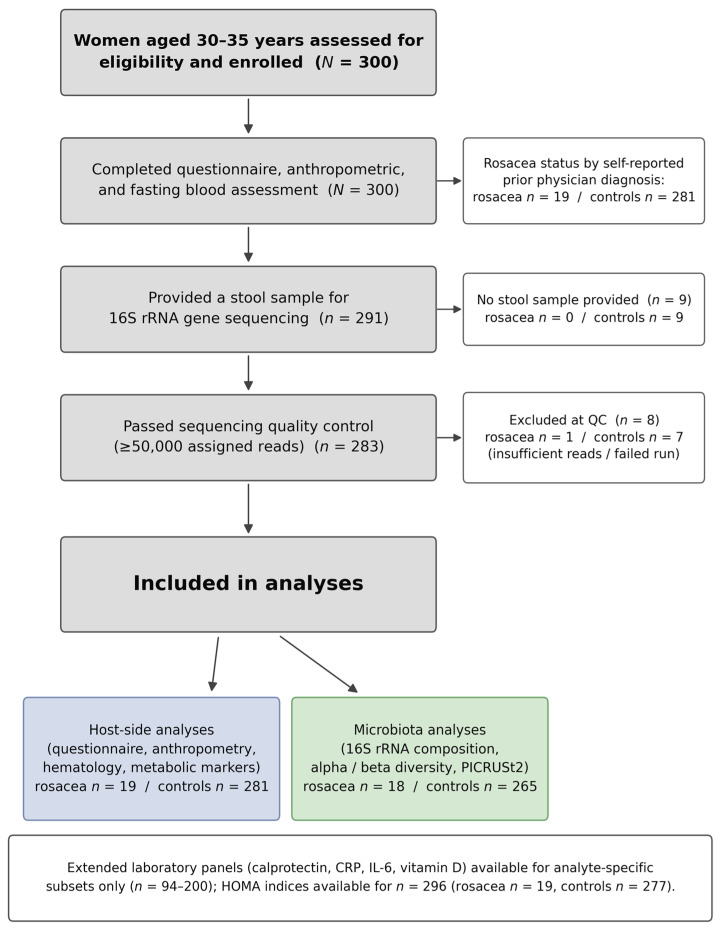
Participant enrollment, exclusions, and sample availability. All 300 enrolled women completed questionnaires and anthropometric and fasting blood assessment. Rosacea status was assigned by self-reported prior physician diagnosis (rosacea *n* = 19; controls *n* = 281). Stool samples were provided by 291 participants; 8 were excluded at sequencing quality control (<50,000 assigned reads or failed run), leaving a microbiota analytic sample of 283 (rosacea *n* = 18; controls *n* = 265). Host-side analyses used all 300 participants; HOMA indices were available for 296 (rosacea *n* = 19; controls *n* = 277); extended laboratory panels were available for analyte-specific subsets (*n* = 94–200), with exact denominators given in the table footnotes.

Stool samples were self-collected using the standardized scientific.pro kit (three separate samples per participant, provided by Biomes NGS GmbH, Wildau, Germany) and stored at −20 °C until processing. DNA was extracted with the QIAamp 96 PowerFecal QIAcube HT Kit (Qiagen, Hilden, Germany) on automated liquid-handling systems ([Hamilton StarLine (Hamilton Company, Reno, NV, USA) & Tecan EVO (Tecan Group Ltd., Männedorf, Switzerland)]). Library preparation followed a modified version of Illumina’s “16S Metagenomic Sequencing Library Preparation” protocol. DNA concentrations were quantified using a fluorescent dye and a BioTek Synergy HTX plate reader (Agilent Technologies, Santa Clara, CA, USA). Sequencing was performed on an Illumina NextSeq 2000 (Illumina, San Diego, CA, USA). Reads shorter than 150 bases were removed, and quality was evaluated using a minimum Phred threshold of 20. Chimeric sequences were identified using vsearch uchime2_ref (v2.7.0) and removed based on comparison with SILVA v138.1. Paired-end reads were merged using PANDAseq, followed by alignment with BLASTn against SILVA (version: 138.1). A minimum of 50,000 assigned reads per sample was required. Identity thresholds for taxonomic levels followed Yarza et al.: phylum 75.0%, class 78.5%, order 82.0%, family 86.5%, genus 94.5%, and species 97.0%. Sequences were clustered at 97% similarity using CD-HIT. Biologically normalized abundances were calculated from clustered sequences using PICRUSt2 with the MetaCyc pathway library. Alpha diversity (Shannon entropy, Chao1, Pielou evenness, Faith’s phylogenetic diversity) was calculated using QIIME 2 and scikit-bio (v0.5.6). Relative abundances were computed at the phylum, family, genus, and species levels. After application of the read-count threshold and removal of samples that failed quality control, microbiota data were available for 283 of the 300 participants (rosacea *n* = 18; controls *n* = 265); the reasons for this reduction are summarized in the enrollment flowchart (Figure 1).

### 2.6. Statistical Analysis

This study follows the STROBE guidelines for cross-sectional studies and the STORMS guidelines for microbiome research reporting (Supplementary Checklists S1 and S2). Continuous variables are reported as median [interquartile range, IQR]. Between-group comparisons used Mann–Whitney U tests; categorical variables used Fisher’s exact test or the chi-squared test. Host-side clinical comparisons (hematological, metabolic, and questionnaire-based) were not corrected for multiple testing, and the corresponding *p*-values are therefore reported and interpreted strictly as nominal, exploratory values with an elevated false-positive risk; they are not described as “significant” in the inferential sense. Alpha diversity was assessed using four complementary indices—Shannon entropy, Chao1 richness, Pielou evenness, and Faith’s phylogenetic diversity—each compared between groups using the Mann–Whitney U test. Beta diversity was assessed using Bray–Curtis dissimilarity with PERMANOVA (9999 permutations) on rosacea status, and stratified PERMANOVA with permutations restricted within BMI tertiles was used as a sensitivity check for residual compositional structure attributable to adiposity. Taxonomic composition was analyzed at four levels—phylum (17 testable taxa), family (119), genus (304), and species (459)—with per-level Benjamini–Hochberg false discovery rate (FDR) correction as the primary adjustment; a global FDR across all 899 features was computed as a sensitivity analysis. Additional analyses included 15 Biome-derived functional modules, 394 PICRUSt2-inferred MetaCyc pathways (with per-level FDR), and four alpha diversity indices. PICRUSt2 pathways are predicted from the same 16S taxonomic abundances and are therefore not independent of the compositional findings. Key genus-level comparisons were repeated using rank-based residuals after adjustment for BMI alone, and were additionally repeated in extended models that adjusted for BMI, sleep quality (SQS), and metabolic markers (HOMA-B, HOMA-IR, fasting insulin) jointly, in order to evaluate whether the host-side covariates that differed between groups attenuated, accounted for, or reversed the microbiota-level associations. A pre-specified sensitivity analysis compared rosacea (*n* = 19) with comorbidity-free controls (*n* = 91) and comorbid controls (*n* = 190) using the Kruskal–Wallis test with post hoc pairwise comparisons.

### 2.7. Handling of Class Imbalance in the Exploratory Classifier

The two groups were severely imbalanced (rosacea *n* = 19 vs. controls *n* = 281; approximately 1:15), and the microbiota subcohort was even smaller on the case side (*n* = 18). We did not interpret this imbalance as a problem to be “solved” by a single technique; rather, we treated it as a fundamental limitation that constrains what any classifier built on these data can legitimately claim. Several complementary measures were taken, and their residual limitations are stated explicitly.

- Algorithm configuration. The exploratory Random Forest classifier (scikit-learn (v1.3); 500 trees, maximum depth 3, minimum 3 samples per leaf) was trained with balanced class weights, which reweight the minority class inversely to its frequency so that the loss function is not dominated by the majority class.
- Performance estimation. Leave-one-out cross-validation (LOOCV) was used as the primary performance estimate because, for a minority class of fewer than 20 observations, k-fold schemes can produce folds containing no cases at all. The area under the ROC curve (AUC) was chosen as the headline metric because, unlike accuracy, it is not inflated by a trivial majority-class prediction; we additionally inspected sensitivity and specificity at the default threshold.
- Resampling-based sensitivity analyses. To verify that the reported performance was not an artifact of a single imbalanced split, we repeated the LOOCV pipeline (i) on 1000 random balanced subsamples in which the controls were down-sampled to the number of cases, and (ii) using SMOTE oversampling of the minority class applied within each cross-validation training fold only. Both procedures yielded AUC distributions whose central tendency was close to the primary LOOCV estimate but with wide confidence intervals (Supplementary Figure S1), confirming that no resampling strategy materially improved—or rescued—discrimination.
- Acknowledged residual limitation. Feature selection was performed on the same dataset used for model evaluation. With only 18–19 cases, fully nested feature selection was not statistically feasible, and we did not claim that any novel statistical method can remove the optimistic bias this introduces. The classifier is therefore presented strictly as a hypothesis-generating, internal-coherence check and not as a diagnostic tool or as evidence of generalizable discrimination.

As an additional, fully independent check, LOOCV-predicted probabilities were correlated (Spearman) with serum calprotectin—an inflammatory biomarker not included in training (*n* = 187). Analyses used Python (v3.11.8; scipy, pandas, scikit-learn, QIIME2, scikit-bio v0.5.6, PICRUSt2).

### 2.8. Use of AI-Assisted Tools

In the interest of full transparency, the authors disclose that a large language model (Claude, Anthropic) was used as an assistive tool during the preparation of this manuscript. Its use was limited to (i) scripting assistance for the statistical analyses in Python (v3.11.8), (ii) literature-search assistance, (iii) language editing and drafting support, (iv) figure-layout assistance, and (v) reference formatting and ordering. The AI tool was not used to generate, analyze, or interpret primary data, and it did not contribute to study design or to the scientific conclusions. All statistical outputs were independently verified by the authors against the underlying data, all citations were checked against primary sources, and all text was reviewed, edited, and approved by the named authors, who take full responsibility for the accuracy and integrity of the work. This disclosure is repeated in the Section 5. in accordance with journal policy.

## 3. Results

The flow of participants from enrollment through to the analytic samples, including the reasons for sample attrition and the analyte-specific denominators, is summarized in Figure 1.

### 3.1. Participant Characteristics, Lifestyle, and Comorbidities

Rosacea was reported by 19 of 300 participants (6.3%). The two groups were comparable in age (median 32 years in both; *p* = 0.417) and height (*p* = 0.937). Women with rosacea had higher body weight (67.0 vs. 63.0 kg; *p* = 0.025) and BMI (23.8 vs. 22.1 kg/m^2^; *p* = 0.037) and reported poorer sleep quality on the SQS (32.0 vs. 29.0, where higher scores indicate poorer sleep; *p* = 0.038). No meaningful differences emerged for dietary quality scores (MDD-W *p* = 0.098; All-5 *p* = 0.114; GDR *p* = 0.608), psychological well-being (WHO-5 *p* = 0.400), mental health quality of life (MHQoL-7D *p* = 0.353), perceived stress (PSS *p* = 0.338), personality traits (all *p* > 0.25), or religiousness (all *p* > 0.19). Smoking prevalence was similar between groups (31.6% vs. 25.6%; *p* = 0.592), while alcohol consumption showed a trend toward a different distribution (χ^2^ = 7.20, df = 3, *p* = 0.066), with a notably higher proportion of non-drinkers in the rosacea group (26.3% vs. 8.2%).

The proportion of women with at least one self-reported comorbidity was 78.9% in the rosacea group versus 67.6% in controls (*p* = 0.445). Migraine stood out as the most prevalent comorbidity in the rosacea group (31.6% vs. 8.5%; OR = 4.94, *p* = 0.007), followed by acne (26.3%), GERD (21.1%), gastritis (21.1%), and endometriosis (21.1%). The mean comorbidity count was higher in the rosacea group, although the difference fell short of nominal significance (2.16 ± 1.86 vs. 1.35 ± 1.27; *p* = 0.056). In summary, rosacea was selectively associated with higher adiposity, poorer sleep, and a higher migraine burden, but not with diet quality, psychological well-being, stress, or personality. As stated in Section 2.6, these host-side comparisons were not corrected for multiple testing and are reported as nominal, exploratory values (Table 1).

### 3.2. Hematological and Metabolic Parameters (Host-Side, Uncorrected)

Among hematological parameters, women with rosacea had a nominally higher erythrocyte concentration (4.60 vs. 4.55 × 10^12^/L; *p* = 0.049), a higher monocyte count (0.57 vs. 0.48 × 10^9^/L; *p* = 0.031), and a lower mean corpuscular hemoglobin concentration (MCHC 328 vs. 332 g/L; *p* = 0.033). By contrast, derived composite inflammatory indices remained entirely non-significant: the neutrophil-to-lymphocyte ratio (NLR 1.60 vs. 1.57; *p* = 0.954), platelet-to-lymphocyte ratio (PLR 123 vs. 126; *p* = 0.789), and systemic immune-inflammation index (SII 418 vs. 404; *p* = 0.994) showed no separation between groups. Other CBC parameters, glucose, the lipid panel, renal function, ferritin, and CRP did not differ significantly, and serum vitamin B12 and vitamin D were similar between groups (Table 2).

**Figure 2:**
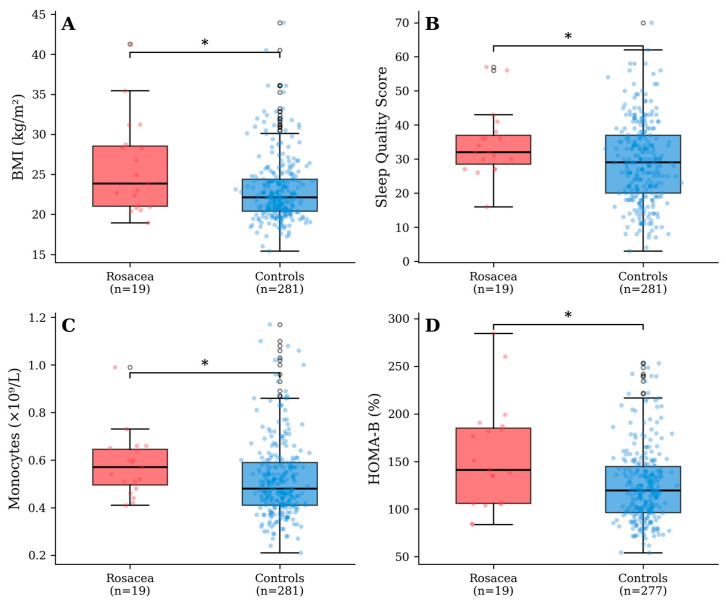
Host-side metabolic and clinical correlates of rosacea (uncorrected for multiple testing). Box plots show (**A**) BMI, (**B**) Sleep Quality Score, (**C**) monocyte count, and (**D**) HOMA-B for the rosacea (in red) and control groups (in blue). Boxes denote the median and interquartile range (IQR); whiskers extend to the most extreme values within 1.5× IQR of the box. Solid jittered points are individual participants within the whisker range; open circles denote statistical outliers (values beyond 1.5× IQR), which were retained in all analyses and are plotted with a distinct symbol only to aid visual identification. In panels (**A**–**C**) the denominators are rosacea *n* = 19 and controls *n* = 281; in panel (**D**) they are rosacea *n* = 19 and controls *n* = 277, because fasting insulin and glucose—required to compute HOMA-B—were missing for four control participants (see Figure 1). * *p* < 0.05, Mann–Whitney U test.

HOMA-B was significantly higher in the rosacea group (141.1 vs. 119.6%; *p* = 0.013), accompanied by trend-level elevations in fasting insulin (78.4 vs. 60.1 pmol/L; *p* = 0.061), HOMA-IR (1.67 vs. 1.26; *p* = 0.065), cortisol (382.8 vs. 344.7 nmol/L; *p* = 0.058), and serum calprotectin (1.22 vs. 0.88 mg/L; *p* = 0.069). The pattern of a nominally elevated HOMA-B alongside a trending HOMA-IR is physiologically coherent: in the natural history of insulin resistance, β-cell compensation typically precedes overt peripheral resistance, with the pancreas upregulating insulin secretion to maintain normoglycemia before HOMA-IR crosses a conventional threshold. We emphasize, however, that these host-side comparisons involved multiple uncorrected tests and that individual *p*-values carry an elevated false-positive risk (Figure 2).

### 3.3. Gut Microbiota Composition (Exploratory, FDR-Negative)

In the microbiota subcohort (rosacea *n* = 18; controls *n* = 265), alpha diversity indices provided a mixed picture. Shannon Entropy showed a trend toward lower values in rosacea (7.20 vs. 7.65; *p* = 0.093), while Chao1 richness, Pielou Evenness, and Faith’s Phylogenetic Diversity did not differ between groups. Beta diversity analysis using Bray–Curtis dissimilarity revealed a non-significant trend toward compositional separation (PERMANOVA: pseudo-F = 1.33, *p* = 0.147, R^2^ = 0.47%), indicating that rosacea explained less than half a percent of overall community variance.

At the phylum level (17 testable phyla), rosacea was nominally associated with higher Verrucomicrobia (0.363% vs. 0.099%; *p* = 0.040) and lower Actinobacteria (1.790% vs. 2.993%; *p* = 0.047), with a trend toward lower Proteobacteria (*p* = 0.077). The dominant phyla Firmicutes and Bacteroidetes did not differ. At the family level (119 testable), seven families reached nominal significance. The most pronounced signal was for *Prevotellaceae*, which was enriched in rosacea with a large fold change (6.244% vs. 0.579%; *p* = 0.007; log2FC = +3.43), followed by depleted *Bifidobacteriaceae* (0.586% vs. 1.926%; *p* = 0.011). Additional enriched families included SRB2 (sulfate-reducing bacteria; *p* = 0.014), *Sporomusaceae* (*p* = 0.015), and *Veillonellaceae* (*p* = 0.033).

**Figure 3:**
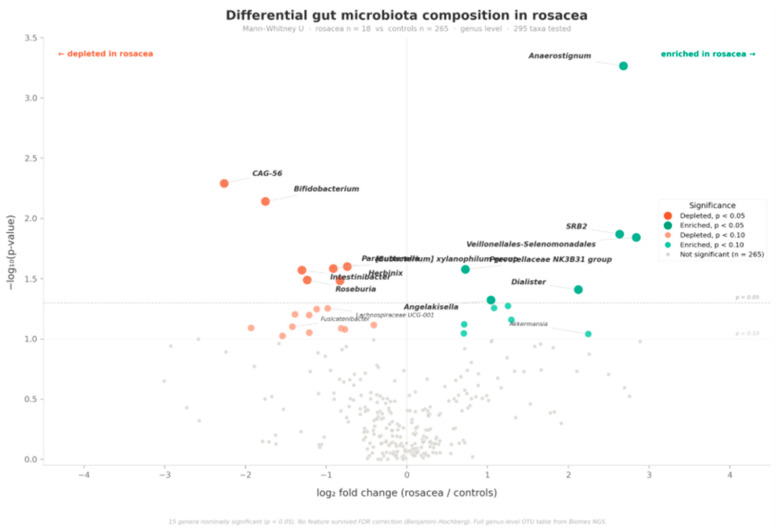
Differential gut microbiota composition in rosacea versus controls at the genus level. Volcano plot in which each point represents one of the 304 testable genera. The *x*-axis shows the log2 fold change in median relative abundance (rosacea/controls) and the *y*-axis shows −log10(*p*-value) from the Mann–Whitney U test (rosacea *n* = 18, controls *n* = 265). Coral points are genera depleted in rosacea and teal points are genera enriched in rosacea; large points denote *p* < 0.05 and medium points 0.05 ≤ *p* < 0.10, while small grey points are non-significant genera (*p* ≥ 0.10). The horizontal dashed line marks the nominal *p* = 0.05 threshold. Genus labels are shown for all nominally significant taxa, with leader lines connecting each label to its point to avoid overlap. No feature survived Benjamini–Hochberg FDR correction (lowest q = 0.165 for *Anaerostignum*).

At the genus level (304 testable genera), 15 reached nominal significance (*p* < 0.05). The lowest *p*-value was observed for *Anaerostignum* (enriched in rosacea; *p* < 0.001; log2FC = +2.68), a *Lachnospiraceae* member with limited prior characterization in clinical cohorts. Among depleted genera, *CAG-56* (*p* = 0.005; also *Lachnospiraceae*), *Bifidobacterium* (0.571 vs. 1.925; *p* = 0.007), [*Eubacterium*] *xylanophilum* group (*p* = 0.025), *Parasutterella* (0.066 vs. 0.125; *p* = 0.026), *Intestinibacter* (*p* = 0.027), *Roseburia* (1.405 vs. 3.307; *p* = 0.033), and *Herbinix* (*p* = 0.033) were all lower in rosacea. Among enriched genera, *Comamonas* (*p* = 0.008), *Prevotellaceae* NK3B31 group (*p* = 0.027), *Dialister* (*p* = 0.039), and *Angelakisella* (*p* = 0.048) were higher. *Akkermansia* showed a trend toward enrichment (0.350 vs. 0.074; *p* = 0.091) (Figure 3).

At the species level (459 testable), the *Bifidobacterium* signal was driven by three species: *B. longum* (*p* = 0.009), *B. angulatum* (*p* = 0.011), and *B. breve* (*p* = 0.012). Other depleted species included *Roseburia* spp. (*p* = 0.024) and *Romboutsia hominis* (*p* = 0.025). Species-level assignments from V3–V4 amplicons carry substantial uncertainty and should be interpreted with caution.

**Figure 4:**
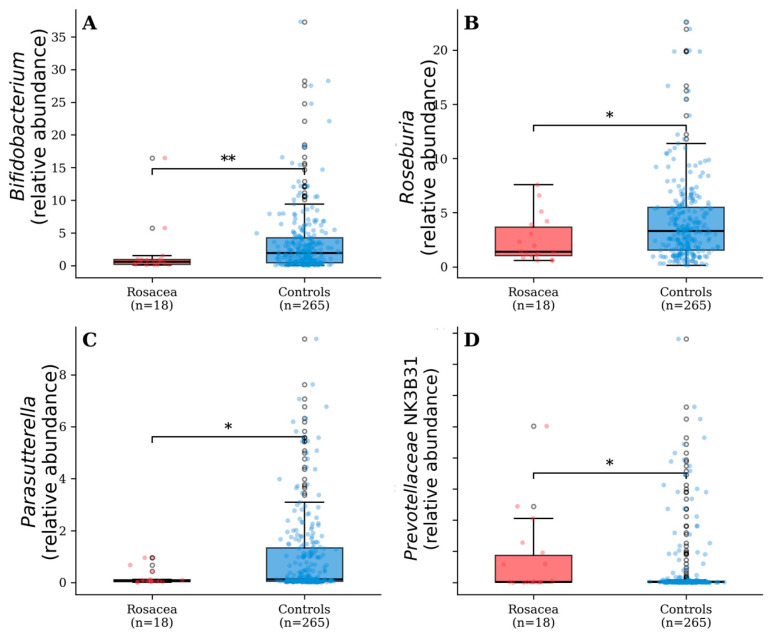
Selected nominally significant gut microbiota genera in rosacea versus controls. Box plots show the relative abundance of (**A**) *Bifidobacterium*, (**B**) *Roseburia*, (**C**) *Parasutterella*, and (**D**) *Prevotellaceae* NK3B31 group (rosacea in red; *n* = 18, controls in blue; *n* = 265). Boxes denote the median and IQR; whiskers extend to the most extreme values within 1.5× IQR. Solid jittered points are individual participants within the whisker range; open circles denote statistical outliers (values beyond 1.5× IQR), which were retained in all analyses and are plotted with a distinct symbol only to aid visual identification. * *p* < 0.05, ** *p* < 0.01, Mann–Whitney U test. None of these features survived FDR correction; the panels are shown to illustrate the direction and spread of the nominal, exploratory signals only.

Critically, no feature survived per-level FDR correction at any taxonomic level (lowest q = 0.165 at genus level for *Anaerostignum*; species q = 0.249; family q = 0.366; phylum q = 0.399). A global FDR across all 899 features yielded a lowest q of 0.244. The total number of nominally significant taxa (48/899 = 5.3%) did not exceed the 5% expected by chance (binomial test *p* = 0.340). The microbiota findings are therefore exploratory and cannot be distinguished from the null hypothesis on the basis of statistical significance alone. Nevertheless, BMI-adjusted analyses confirmed that all key genera remained nominally significant after rank-based regression adjustment (all BMI–taxon |ρ| < 0.09), and the concordance within individual taxonomic lineages—Actinobacteria at the phylum level mapping onto *Bifidobacteriaceae* at the family level and *Bifidobacterium* at the genus level, and Verrucomicrobia mapping onto *Akkermansia*—provides internal coherence that may warrant targeted follow-up in adequately powered studies (Table 3; Figure 4).

PICRUSt2 pathway analysis identified 10 nominally significant MetaCyc pathways (all enriched in rosacea), including three cobalamin biosynthesis pathways (all *p* < 0.046) and two tryptophan/kynurenine pathways (both *p* = 0.049). No pathway survived FDR correction (m = 394; lowest q = 0.768). Because PICRUSt2 pathways are predicted from the same 16S data, they are not independent of the compositional findings and should be viewed as complementary annotations of the same underlying signal rather than as independent validation (Supplementary Table S2; Supplementary Results).

### 3.4. Sensitivity Analysis

**Figure 5:**
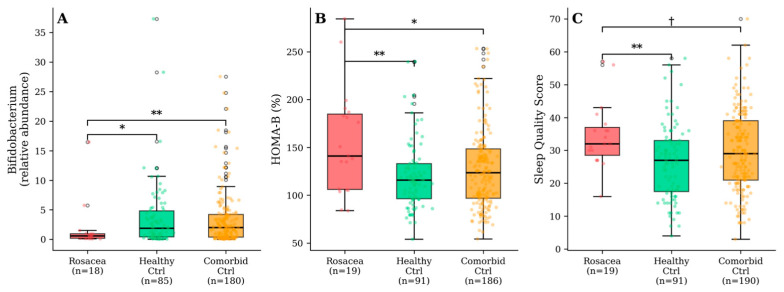
Sensitivity analysis: rosacea versus healthy and comorbid control subgroups. Box plots show (**A**) *Bifidobacterium* relative abundance, (**B**) HOMA-B, and (**C**) Sleep Quality Score across the three groups. For the microbiota panel (**A**) the denominators are rosacea in red; *n* = 18, healthy controls in green; *n* = 85, and comorbid controls in orange; *n* = 180; for the host-side panels (**B**,**C**) they are rosacea *n* = 19, healthy controls *n* = 91, and comorbid controls *n* = 190 (the microbiota denominators are smaller because of sequencing quality-control exclusions; see Figure 1). Boxes denote the median and IQR; whiskers extend to within 1.5× IQR; solid jittered points are individual participants within the whisker range; open circles denote retained statistical outliers. Significance markers refer to pairwise post hoc comparisons: * *p* < 0.05, ** *p* < 0.01, † 0.05 ≤ *p* < 0.10 (trend, non-inferential). All comparisons are nominal and were not FDR-corrected.

To evaluate whether the observed differences were driven by general comorbidity burden rather than rosacea per se, a three-group comparison was performed: all rosacea participants (*n* = 19) versus comorbidity-free controls (*n* = 91) versus controls with at least one comorbidity (*n* = 190). *Bifidobacterium* remained nominally lower in rosacea compared to both comorbidity-free controls (*p* = 0.015) and comorbid controls (*p* = 0.007), while the two control subgroups did not differ from each other (*p* = 0.939), suggesting that the depletion is not a generic comorbidity effect. HOMA-B was nominally higher in rosacea versus both control subgroups (*p* = 0.008 vs. healthy; *p* = 0.024 vs. comorbid). Sleep quality was worse in rosacea versus healthy controls (*p* = 0.006) (Figure 5).

*Roseburia* was nominally lower in rosacea versus comorbid controls (*p* = 0.024) but only trended versus healthy controls (*p* = 0.093). Calprotectin reached nominal significance when comparing rosacea to comorbidity-free controls (*p* = 0.026). These comparisons were not FDR-corrected and should be interpreted cautiously (Supplementary Table S1).

### 3.5. Exploratory Classification

Individual genera showed moderate discriminatory power in single-feature ROC analysis: *Anaerostignum* AUC = 0.717, *CAG-56* AUC = 0.697, and *Bifidobacterium* AUC = 0.689. The integrated Random Forest model (five genera plus HOMA-B and cortisol) achieved the best cross-validated performance: LOOCV AUC = 0.785 and 5-fold CV AUC = 0.804 ± 0.105, compared with LOOCV AUC = 0.748 for the microbiota-only model. The most informative features were *Bifidobacterium* (Gini importance 0.20), *CAG-56* (0.17), and *Anaerostignum* (0.16), followed by HOMA-B (0.14) and cortisol (0.11). As detailed in Section 2.7, the apparent (resubstitution) AUC of 0.989 substantially exceeded all cross-validated estimates, confirming pronounced overfitting at *n* = 18, and feature selection performed on the same data further inflates cross-validated performance. The resampling-based sensitivity analyses (balanced down-sampling and within-fold SMOTE) did not materially change the LOOCV AUC, indicating that the imbalance handling neither rescued nor exaggerated discrimination. LOOCV-predicted probabilities showed a weak but nominally significant correlation with serum calprotectin (ρ = 0.234, *p* = 0.001, *n* = 187), while the microbiota-only model showed only a trend (ρ = 0.136, *p* = 0.064). The classifier results should be regarded as hypothesis-generating and do not provide reliable estimates of out-of-sample discrimination (Supplementary Figure S1; Supplementary Methods). The broader pattern of cross-domain associations underlying these exploratory analyses, between microbiota taxa, functional modules, and systemic metabolic and inflammatory markers across the full cohort (*n* = 283) is summarised as a Spearman correlation matrix in Supplementary Figure S2 (see Supplementary Results).

## 4. Discussion

This study examined gut microbiota composition alongside metabolic, immune, and lifestyle parameters in a narrowly age-defined cohort of young women with self-reported rosacea. The findings fall into two categories that differ in their statistical robustness and should be interpreted accordingly. On the host side, several metabolic and immune parameters—notably HOMA-B and monocyte count—reached nominal significance in uncorrected comparisons, and HOMA-B survived a pre-specified sensitivity analysis across three subgroups. On the microbiome side, although nominally significant differences were observed at multiple taxonomic levels, no feature survived FDR correction, and the overall number of nominally significant taxa did not exceed chance expectation. The microbiota findings are therefore exploratory and are presented as hypothesis-generating observations that require independent confirmation.

### 4.1. Exploratory Microbiota Findings in the Context of Existing Literature

Alpha diversity has been inconsistently associated with rosacea across the nine published fecal microbiota studies. Nam et al. found no difference in alpha or beta diversity in their Korean cohort [19]. Chen et al. observed reduced richness (Chao1) but not Shannon diversity in their Taiwanese patients [20]. Guertler et al., in the largest study to date, reported method-dependent alpha diversity results [23], while Joura et al. found limited stool-level differences despite distinct skin microbiota profiles [25]. In our cohort, Shannon entropy showed a trend (*p* = 0.093) while other alpha metrics did not differ, and PERMANOVA did not reach significance (*p* = 0.147). Taken together, the evidence across studies suggests that global diversity indices are not robust discriminators of rosacea-associated microbiota, and that compositional analysis at lower taxonomic levels may be more informative—although in our case such analyses also did not survive multiple-testing correction.

Among the nominally significant genera in our cohort, the depletion of *Roseburia* (*p* = 0.033) is of particular interest because it is directionally concordant with two independent studies. Chen et al. reported reduced *Roseburia* in their Taiwanese cohort of 11 patients [20], and Zhao et al. confirmed the same finding in a Beijing cohort using whole-metagenome sequencing—a different methodology that provides higher taxonomic resolution [28]. *Roseburia* is among the most abundant butyrate-producing genera in the human colon, and its depletion has been linked to impaired gut barrier integrity across a range of inflammatory and metabolic conditions [34]. Butyrate serves as the primary energy source for colonocytes, maintains tight junction integrity through upregulation of claudin-1 and suppression of the permeability-promoting claudin-2, and exerts broad anti-inflammatory effects through inhibition of class I and II histone deacetylases (HDACs), promoting histone H3 and H4 acetylation that suppresses NF-κB-driven cytokine production in macrophages [35,36,37,38]. Butyrate also signals through GPR109A on intestinal epithelial and immune cells, further reinforcing barrier function and promoting regulatory T-cell differentiation [35]. However, the concordance of *Roseburia* depletion across three small studies should not be over-interpreted: all three datasets involved small rosacea groups (*n* = 11–18), and selective reporting of depleted butyrate producers—a widely recognized “gut health” narrative—cannot be excluded as a contributor to apparent cross-study consistency.

The nominal depletion of *Bifidobacterium* (*p* = 0.007) presents an instructive contrast with the existing literature. Chen et al. reported *Bifidobacterium* enrichment in their Taiwanese rosacea patients—the opposite direction from our finding [20]. Several factors may explain this discrepancy. Our cohort consists exclusively of young European women aged 30–35 years, whereas Chen et al. studied predominantly older patients (mean age 53 years) in East Asia, and age is one of the strongest determinants of *Bifidobacterium* abundance, which declines substantially after the fourth decade of life. Dietary patterns, host genetics, and the phenotypic spectrum of rosacea may further contribute to population-level differences. *Bifidobacterium* is a central immunomodulatory genus that produces acetate and lactate, promotes intestinal barrier integrity through tight junction protein upregulation, and supports regulatory T-cell differentiation [39]. The observation that the depletion in our cohort involved three species—*B. longum*, *B. angulatum*, and *B. breve*—has not been reported in any previous rosacea study, although species assignments from V3–V4 amplicons should be treated with considerable caution.

The nominal depletion of *Parasutterella* (*p* = 0.026) is novel to the rosacea literature. *Parasutterella* is a core member of the healthy gut microbiota that has been linked to bile acid metabolism, tryptophan homeostasis, and intestinal immune regulation [40]. Its depletion could, in principle, contribute to altered bile acid signaling and reduced tryptophan-derived anti-inflammatory metabolites, a notion that is consistent with the nominal enrichment of PICRUSt2-predicted tryptophan/kynurenine pathways. However, this chain of reasoning rests on multiple FDR-negative observations, each individually indistinguishable from chance, and should be treated accordingly.

The enrichment of *Anaerostignum*—the strongest genus-level signal (q = 0.165)—deserves mention precisely because it has limited prior characterization in clinical cohorts. As a *Lachnospiraceae* member, it belongs to the same family as *Roseburia* and other butyrate producers, but its functional role in the human gut remains poorly understood. The trend toward higher *Akkermansia* (*p* = 0.091, a non-inferential trend) may represent a compensatory response to barrier disruption, as *Akkermansia muciniphila* is known to thrive on mucin and may proliferate in the context of altered mucus-layer dynamics [41]. Moreno-Arrones et al. also noted shifts in mucin-associated taxa in their Spanish pilot, although the specific genera differed [21].

The PICRUSt2-predicted enrichment of cobalamin biosynthesis pathways (three pathways, all *p* < 0.046) extends a finding originally reported by Chen et al. using the same predictive approach [20]. Two tryptophan/kynurenine pathways were also nominally enriched (both *p* = 0.049). The kynurenine pathway diverts tryptophan toward neuroactive and immunomodulatory metabolites—including quinolinic acid and kynurenic acid—at the expense of serotonin synthesis [42], a mechanism increasingly implicated in neuroinflammatory conditions. However, because PICRUSt2 pathways are inferred from the same 16S data, they provide no independent confirmation of the compositional findings, and none survived FDR correction.

### 4.2. Host-Side Metabolic and Immune Findings

In contrast to the FDR-negative microbiota results, several host-side parameters showed more consistent patterns, although these comparisons were also uncorrected for multiple testing. The finding of nominally elevated HOMA-B (*p* = 0.013) is of interest because it survived the three-group sensitivity analysis, remaining nominally significant versus both comorbidity-free controls (*p* = 0.008) and comorbid controls (*p* = 0.024). In the natural history of insulin resistance, β-cell compensation precedes overt peripheral resistance: the pancreas upregulates insulin secretion to maintain normoglycemia before HOMA-IR crosses a conventional threshold. Our data appear to capture this early phase, with concordant trend-level elevations in fasting insulin (*p* = 0.061) and HOMA-IR (*p* = 0.065). Akbaba et al. reported elevated HOMA-IR in Turkish rosacea patients [32], and Sodagar et al. confirmed a meta-analytic association between metabolic syndrome and rosacea across multiple cohorts [31], lending plausibility to our observation. However, confounding by BMI cannot be fully excluded despite the sensitivity analysis, and other studies have reported weaker or null metabolic associations that likely reflect differences in age, sex composition, and rosacea phenotype.

The isolated monocyte elevation (*p* = 0.031) alongside entirely non-significant NLR (*p* = 0.954), PLR (*p* = 0.789), and SII (*p* = 0.994) presents a notable discrepancy with Karaosmanoglu et al., who reported elevated NLR and PLR in a Turkish cohort with broader age and sex composition [30]. In our narrowly defined cohort, the selective monocyte signal—rather than a broad neutrophil-driven inflammatory response—may represent a different immune signature in young women with rosacea, although this interpretation rests on a single uncorrected comparison. The trending serum calprotectin (*p* = 0.069)—a calcium-binding protein released predominantly by activated monocytes—is directionally concordant and reached nominal significance versus comorbidity-free controls in the sensitivity analysis (*p* = 0.026), but did not reach significance in the primary two-group comparison.

### 4.3. Integrative Interpretation

The co-occurrence of nominally depleted SCFA-producing taxa, elevated HOMA-B, monocytosis, and poorer sleep quality in the rosacea group is compatible with a gut–skin axis model in which gut dysbiosis, metabolic perturbation, and systemic inflammation interact to contribute to rosacea pathogenesis. However, several important caveats prevent us from interpreting this pattern as evidence for such a model. First, the microbiota component rests entirely on FDR-negative, nominally significant findings that, as an ensemble, do not exceed chance expectation. Second, the host-side findings, while nominally more robust, were not corrected for multiple testing across the many parameters examined. Third, the cross-sectional design cannot distinguish cause from consequence or shared confounding by unmeasured factors.

The nominally higher migraine prevalence in the rosacea group (OR = 4.94, *p* = 0.007) is consistent with shared trigeminal nerve activation and neurogenic inflammation, a pathophysiological overlap documented in several population-based studies [16].

In this context, the nominal enrichment of PICRUSt2-predicted tryptophan/kynurenine pathways acquires particular interest: enhanced tryptophan catabolism through the kynurenine pathway generates neuroactive metabolites at the expense of serotonin synthesis [42], providing a potential mechanistic bridge between gut composition and neuroimmune features. However, this chain of reasoning remains speculative, given the FDR-negative status and the indirect nature of PICRUSt2 annotations.

The trending cortisol elevation (*p* = 0.058) in the absence of elevated perceived stress (PSS *p* = 0.338) would be consistent with HPA-axis activation by gut-derived inflammatory signals rather than subjective psychological distress, but could equally reflect unmeasured confounders, diurnal variability, or sample-size limitations. Poorer sleep quality (*p* = 0.038) adds to a growing literature linking sleep disturbance with inflammatory skin conditions; sleep deprivation is known to increase cortisol, promote insulin resistance, and alter gut microbiota, including reduction in *Bifidobacterium* [43], but the causal direction of this association cannot be established in a cross-sectional design.

The exploratory classifier’s weak correlation with calprotectin (ρ = 0.234)—a biomarker not included in the model—provides a tentative suggestion that the combined microbiota–metabolic signature may capture a dimension of systemic inflammatory burden beyond its individual input features. This observation is hypothesis-generating and would require validation in an independent cohort before any further interpretation is warranted. Mendelian randomization data suggesting a causal trajectory from gut microbiota toward rosacea [33] and the therapeutic efficacy of SIBO eradication with rifaximin [14] provide broader contextual support for the gut–skin axis concept, but our study does not add to the causal evidence base.

### 4.4. Strengths and Limitations

Strengths of this study include the narrowly age-defined, all-female cohort (30–35 years), which minimizes confounding by age and sex—the two major sources of microbiota variability that have hampered interpretation of previous rosacea studies, which included patients ranging from 20 to 80 years with variable sex ratios. The integrative approach combining 16S rRNA sequencing with comprehensive HOMA-based metabolic profiling, CBC-derived immune markers, and validated lifestyle questionnaires is unique in the rosacea literature. The three-group sensitivity analysis comparing rosacea with both comorbidity-free and comorbid control subgroups is a methodological contribution not employed in any previous rosacea–microbiota study. Our rosacea microbiota sample (*n* = 18) is comparable in size to the two East Asian NGS studies (Nam et al., *n* = 12; Chen et al., *n* = 11) and represents, together with the German cohort of Guertler et al. (*n* = 54), the Spanish pilot of Moreno-Arrones et al. (*n* = 15), and the Turkish cohort of Yilmaz et al., one of the few Western populations examined [19,20,21,22,23]. Antibiotic exposure within three months was an explicit exclusion criterion, reducing one major source of microbiome confounding that was not uniformly controlled in previous studies.

The study limitations are substantial and constrain interpretation of the findings. The most consequential limitation relates to case ascertainment. Rosacea was identified by self-report of a prior physician diagnosis, without independent dermatological verification, clinical examination, or phenotype subtyping at the time of study enrollment, as detailed in Section 2.2. This may introduce misclassification bias in both directions: some women with rosacea may have been misclassified as controls (reducing sensitivity), and some controls may have incorrectly reported a rosacea diagnosis (reducing specificity).

The substantial group imbalance (*n* = 19 vs. *n* = 281) further limits statistical power and renders all analyses susceptible to outlier effects. In addition, the use of 16S rRNA V3–V4 amplicon sequencing limits taxonomic resolution to the genus level and provides only predicted (PICRUSt2-inferred), rather than directly measured, functional profiles.

#### 4.4.1. Unmeasured Microbiome Confounders

Several microbiome confounders of established importance were not captured, and this substantially limits the interpretability of the microbiota findings: (i) probiotic and prebiotic supplementation, which directly alters the abundance of key taxa of interest, including *Bifidobacterium* and *Roseburia*; (ii) proton-pump inhibitor therapy, which is known to substantially reshape gut microbiota composition and may be more prevalent in the rosacea group given the higher GERD comorbidity (21.1%); (iii) metformin use, which profoundly alters gut microbiota, including enrichment of *Akkermansia* and modulation of SCFA-producing genera [44]; (iv) current dermatologic treatment for rosacea, including topical or oral agents with potential antimicrobial effects on the gut; (v) stool consistency (Bristol Stool Scale), a major determinant of microbiota composition and transit time that was not recorded; (vi) hormonal contraceptive status; and (vii) recent non-antibiotic medication use. The absence of these data means that the observed microbiota differences could be partly or fully attributable to unmeasured confounders rather than to rosacea itself.

#### 4.4.2. Statistical and Generalizability Constraints

Host-side clinical comparisons were not corrected for multiple testing across the many parameters examined, and individual *p*-values carry an elevated false-positive risk. Extended laboratory panels were available only for a subset of participants because of budgetary constraints, limiting the sample size for calprotectin, CRP, IL-6, and vitamin D comparisons. The all-female, narrow-age design, while a strength for internal validity, limits generalizability to male patients, other age groups, and non-European populations.

### 4.5. Conclusions

In this cohort of young Croatian women with self-reported rosacea, host-side metabolic markers—particularly HOMA-B—showed nominally significant differences that survived a pre-specified sensitivity analysis, suggesting a possible metabolic dimension to rosacea in this demographic that merits further investigation. Fecal microbiota analyses identified nominally significant differences at multiple taxonomic levels, including depletion of *Bifidobacterium* (three species), *Roseburia*, and *Parasutterella*, and enrichment of *Anaerostignum*. However, no feature survived FDR correction, and the overall number of nominally significant taxa did not exceed chance expectation.

The pattern of results is compatible with the gut–skin axis hypothesis but is equally consistent with confounding by unmeasured factors, including medications, dietary supplements, stool consistency, and hormonal status. The nominal depletion of *Roseburia* is directionally concordant with Taiwanese and Chinese cohorts, while the multi-species *Bifidobacterium* depletion and *Anaerostignum* enrichment have not been previously reported. These findings should therefore be viewed strictly as hypothesis-generating. Confirmation in adequately powered cohorts with clinical rosacea verification, comprehensive confounder capture (including medication use, stool consistency, and hormonal status), pre-registered analytical plans, and whole-genome shotgun metagenomics is required. Only if these associations are independently replicated would it become appropriate to consider them as a basis for future stratification of rosacea patients or for the design of gut-directed intervention studies.

## 5. Declarations

Authorship: All named authors meet the International Committee of Medical Journal Editors (ICMJE) criteria for authorship of this article, take responsibility for the integrity of the work as a whole, and have given their approval for this version to be published.

## Figures and Tables

**Table 1:** Demographic, lifestyle, personality, and comorbidity characteristics of participants.

Variable	Rosacea (*n* = 19)	Controls (*n* = 281)	*p*-Value
***Demographics and anthropometrics***			
Age, years	32.0 [31.0–33.5]	32.0 [30.0–33.0]	0.417
Body weight, kg	67.0 [61.5–82.5]	63.0 [57.0–70.0]	**0.025**
Height, cm	168.0 [164.5–172.5]	168.0 [164.0–173.0]	0.937
BMI, kg/m^2^	23.8 [21.0–28.5]	22.1 [20.4–24.4]	**0.037**
***Dietary quality***			
Diet Diversity Score (0–10)	6.0 [4.5–6.0]	6.0 [5.0–7.0]	0.098
All-5 Diet Quality (0–5)	4.0 [4.0–4.0]	4.0 [4.0–5.0]	0.114
NCD-Protect Score (0–9)	3.0 [2.0–4.0]	4.0 [3.0–5.0]	0.231
NCD-Risk Score (0–9)	2.0 [1.0–3.0]	2.0 [1.0–4.0]	0.797
GDR Score (0–18)	10.0 [9.0–11.0]	10.0 [9.0–12.0]	0.608
***Psychological well-being***			
WHO-5 (0–25)	15.0 [11.0–16.0]	15.0 [12.0–17.0]	0.400
MHQoL-7D (0–21)	14.0 [12.5–16.5]	15.0 [14.0–17.0]	0.353
MHQoL-VAS (0–10)	7.0 [6.0–8.0]	7.0 [7.0–8.0]	0.521
SQS (0–84)	32.0 [28.5–37.0]	29.0 [20.0–37.0]	**0.038**
PSS (0–40)	19.0 [17.0–23.5]	18.0 [15.0–23.0]	0.338
***Personality, TIPI (1–7)***			
Extraversion	4.5 [3.2–5.8]	5.0 [3.5–6.0]	0.307
Agreeableness	4.0 [3.5–4.5]	4.0 [3.0–4.5]	0.250
Conscientiousness	6.0 [5.5–6.5]	6.0 [5.0–6.5]	0.571
Emotional Stability	5.0 [3.2–5.5]	4.5 [3.5–5.5]	0.830
Openness	6.0 [5.0–6.5]	6.0 [5.0–6.5]	0.419
***Religiousness***			
Organizational (ORA, 1–6)	2.0 [1.5–4.0]	2.0 [1.0–3.0]	0.194
Non-organizational (NORA, 1–6)	1.0 [1.0–4.0]	1.0 [1.0–4.0]	0.819
Intrinsic (IR, 1–5)	3.0 [1.8–4.0]	3.0 [1.3–4.0]	0.631
***Lifestyle and comorbidities***			
Current smoker, *n* (%)	6 (31.6)	72 (25.6)	0.592
Non-drinker, *n* (%)	5 (26.3)	23 (8.2)	0.066
≥1 comorbidity, *n* (%)	15 (78.9)	190 (67.6)	0.445
Comorbidity count, mean ± SD	2.16 ± 1.86	1.35 ± 1.27	0.056
Migraine, *n* (%)	6 (31.6)	24 (8.5)	**0.007**
Acne, *n* (%)	5 (26.3)	—	—
GERD, *n* (%)	4 (21.1)	—	—
Gastritis, *n* (%)	4 (21.1)	—	—
Endometriosis, *n* (%)	4 (21.1)	—	—

Data are presented as median [IQR] unless otherwise indicated. Between-group comparisons used the Mann–Whitney *U* test (continuous variables) or Fisher’s exact/chi-squared test (categorical variables, as appropriate). Bold *p*-values indicate nominal significance (*p* < 0.05). These comparisons were not corrected for multiple testing and are exploratory. Acne (acne vulgaris), GERD, gastritis and endometriosis frequencies are reported for the rosacea group only to characterise the most prevalent comorbidities in this subgroup; by definition, controls did not carry any of these diagnoses, so no between-group statistics are reported for these individual conditions. Abbreviations: WHO, World Health Organisation; VAS, Visual Analogue Scale; ORA, organizational religious activity; NORA, non-organizational religious activity.

**Table 2:** Hematological, biochemical, and metabolic parameters (host-side, uncorrected).

Variable	Rosacea (*n* = 19)	Controls (*n* = 281)	*p*-Value
***Complete blood count***			
Leukocytes (×10^9^/L)	6.20 [5.75–7.55]	6.10 [5.10–7.30]	0.457
Erythrocytes (×10^12^/L)	4.60 [4.54–4.87]	4.55 [4.33–4.76]	**0.049**
Hemoglobin (g/L)	136 [132–143]	136 [130–142]	0.475
Hematocrit (L/L)	0.41 [0.40–0.43]	0.41 [0.39–0.42]	0.112
MCV (fL)	89.8 [86.9–90.7]	90.1 [87.6–92.2]	0.320
MCH (pg)	29.2 [28.3–30.1]	30.0 [29.0–30.8]	0.059
MCHC (g/L)	328 [324–333]	332 [327–336]	**0.033**
RDW (%)	12.8 [12.3–13.4]	12.6 [12.2–13.0]	0.229
Platelets (×10^9^/L)	262 [234–309]	265 [227–305]	0.692
MPV (fL)	10.1 [9.5–10.5]	10.0 [9.5–10.5]	0.965
Neutrophils (×10^9^/L)	3.44 [2.72–4.64]	3.20 [2.61–4.14]	0.652
Lymphocytes (×10^9^/L)	2.18 [1.77–2.64]	2.09 [1.74–2.44]	0.461
Monocytes (×10^9^/L)	0.57 [0.49–0.65]	0.48 [0.41–0.59]	**0.031**
Eosinophils (×10^9^/L)	0.12 [0.10–0.20]	0.14 [0.10–0.24]	0.406
Basophils (×10^9^/L)	0.03 [0.02–0.04]	0.03 [0.02–0.04]	0.882
***Derived inflammatory indices***			
NLR	1.60 [1.23–2.09]	1.57 [1.23–2.03]	0.954
PLR	123 [96–170]	126 [103–154]	0.789
SII	418 [253–625]	404 [309–553]	0.994
***Biochemistry***			
Glucose (mmol/L)	4.80 [4.75–5.00]	4.80 [4.60–5.10]	0.777
Total cholesterol (mmol/L) ᵃ	5.35 [4.65–6.20]	4.60 [4.20–5.07]	0.140
Triglycerides (mmol/L) ᵃ	0.60 [0.50–0.78]	0.60 [0.50–0.88]	1.000
HDL cholesterol (mmol/L) ᵃ	1.45 [1.32–1.80]	1.50 [1.30–1.70]	0.896
LDL cholesterol (mmol/L) ᵃ	3.00 [2.70–3.45]	2.80 [2.50–3.50]	0.452
Vitamin B12 (pmol/L)	347 [282–428]	362 [291–493]	0.516
Folic acid (nmol/L) ᵃ	15.4 [13.1–17.7]	15.8 [12.5–23.0]	0.591
Vitamin D (nmol/L) ᵃ	71.6 [42.1–81.1]	63.3 [40.5–84.2]	0.655
***Metabolic and hormonal markers***			
Fasting insulin (pmol/L)	78.4 [48.0–106.2]	60.1 [44.2–78.9]	0.061
HOMA-B (%)	141.1 [106.0–184.9]	119.6 [96.4–144.7]	**0.013**
HOMA-S (%)	60.0 [45.4–98.4]	79.4 [59.4–108.9]	0.065
HOMA-IR	1.67 [1.02–2.21]	1.26 [0.92–1.67]	0.065
Cortisol (nmol/L)	382.8 [348.4–477.4]	344.7 [296.5–411.8]	0.058
***Inflammatory biomarkers (subset)***			
Serum calprotectin (mg/L) ᵃ	1.22 [0.84–1.36]	0.87 [0.59–1.18]	0.069

Data are presented as median [IQR]. Between-group comparisons used the Mann–Whitney U test. Bold *p*-values indicate nominal significance (*p* < 0.05). These comparisons were not corrected for multiple testing and are exploratory. ᵃ Extended panels (lipids, vitamin D, folic acid, calprotectin) were available for subsets of participants owing to budgetary constraints: total cholesterol, triglycerides, HDL (high-density lipoprotein), LDL (low-density lipoprotein), and folic acid *n* = 94–140; vitamin D *n* = 150; calprotectin *n* = 200 (rosacea *n* = 13, controls *n* = 187). HOMA indices were available for *n* = 296 (rosacea *n* = 19, controls *n* = 277). HOMA-B, homeostatic model assessment of β-cell function; HOMA-S, insulin sensitivity; HOMA-IR, insulin resistance; MCV, mean corpuscular volume; MCH, mean corpuscular hemoglobin; MCHC, mean corpuscular hemoglobin concentration; MPV, mean platelet volume; NLR, neutrophil-to-lymphocyte ratio; PLR, platelet-to-lymphocyte ratio; RDW, red cell distribution width; SII, systemic immune-inflammation index.

**Table 3:** Nominally significant gut microbiota features (*p* < 0.05). No feature survived FDR correction (all q > 0.05).

Feature	Level	Rosacea (*n* = 18)	Controls (*n* = 265)	*p*	q ᵃ
***Phylum level***					
Verrucomicrobia	Phylum	0.363 [0.042–2.397]	0.099 [0.025–0.537]	0.040	0.399
Actinobacteria	Phylum	1.790 [0.640–3.080]	2.993 [1.200–6.550]	0.047	0.399
***Family level***					
*Prevotellaceae*	Family	6.244 [0.120–12.5]	0.579 [0.080–2.100]	0.007	0.366
*Bifidobacteriaceae*	Family	0.586 [0.300–1.200]	1.926 [0.500–5.100]	0.011	0.366
***Genus level***					
*Anaerostignum*	Genus	0.177 [0.070–0.890]	0.026 [0.000–0.088]	<0.001	0.165
*CAG-56*	Genus	0.000 [0.000–0.016]	0.028 [0.000–0.120]	0.005	0.412
*Bifidobacterium*	Genus	0.571 [0.189–0.926]	1.925 [0.408–4.220]	0.007	0.412
*Comamonas*	Genus	0.000 [0.000–0.008]	0.000 [0.000–0.000]	0.008	0.412
[Eubacterium] xylanophilum grp	Genus	0.007 [0.000–0.061]	0.083 [0.000–0.241]	0.025	0.463
*Parasutterella*	Genus	0.066 [0.041–0.115]	0.125 [0.053–1.331]	0.026	0.463
Prevotellaceae NK3B31 grp	Genus	0.111 [0.071–4.324]	0.067 [0.024–0.170]	0.027	0.463
*Intestinibacter*	Genus	0.066 [0.014–0.118]	0.152 [0.047–0.362]	0.027	0.463
*Roseburia*	Genus	1.405 [1.023–3.679]	3.307 [1.529–5.505]	0.033	0.463
*Herbinix*	Genus	0.000 [0.000–0.000]	0.000 [0.000–0.009]	0.033	0.463
*Dialister*	Genus	1.191 [0.431–2.129]	0.202 [0.042–1.276]	0.039	0.493
*Angelakisella*	Genus	0.000 [0.000–0.014]	0.000 [0.000–0.000]	0.048	0.536
***Functional module***					
Vitamin B12 production ᵇ	Module	32.800 [25.1–47.6]	27.170 [20.0–36.3]	0.048	0.549

Values are median [IQR] of relative abundance (%). Mann–Whitney U test. Only features reaching nominal significance (*p* < 0.05) are listed. ᵃ Benjamini–Hochberg FDR correction within each taxonomic level (17 phyla, 119 families, 304 genera, 459 species). No feature reached q < 0.05. Global FDR across all 899 features: lowest q = 0.244. Total nominally significant taxa: 48/899 (5.3%); binomial test versus the 5% null: *p* = 0.340. ᵇ Biome-derived proprietary functional module. Note on trend-level features: in response to reviewer feedback, features with 0.05 ≤ *p* < 0.10 (e.g., Shannon entropy *p* = 0.093; Fusicatenibacter *p* = 0.079; Akkermansia *p* = 0.091) are deliberately excluded from this table because *p*-values in that range are not statistically meaningful and were not interpreted inferentially.

## Data Availability

The 16S rRNA gene sequencing data generated at the Gut Microbiome Center laboratory are not publicly available because the microbiome data are currently being used in the preparation of a patent application. Public deposition is precluded at this stage for legal and intellectual property reasons. Data are available from the corresponding author upon justifiable request, subject to ethical approval and a data sharing agreement.

## Supplementary Materials

The following supporting information can be downloaded at https://www.mdpi.com/article/10.3390/jcm15114130/s1. Supplementary Table S1: Three-group sensitivity analysis; Supplementary Table S2: Full PICRUSt2 MetaCyc pathway results (394 pathways); Supplementary Methods: Classifier specifications; Supplementary Figure S1: Exploratory integrated microbiota–metabolic classifier (hypothesis-generating); Supplementary Results: PICRUSt2 analysis; Correlation analyses; Biomes functional modules; Supplementary Figure S2: Spearman correlation matrix; Supplementary Checklist S1: STROBE Checklist [45]; Supplementary Checklist S2: STORMS Checklist [46].
