# Supplementary material for: Gut Microbiota, Metabolic Markers, and Systemic Inflammation in Young Women with Self-Reported Rosacea: An Exploratory Cross-Sectional Study
Source: J Clin Med. 2026 May 27;15(11):4130. doi: 10.3390/jcm15114130 (PMC13258242; doi:10.3390/jcm15114130)
Supplement: Supplementary file 1 [file jcm-15-04130-s001.zip › jcm-4302632-supplementary.pdf]

SUPPLEMENTARY MATERIAL

Gut Microbiota, Metabolic Markers, and Systemic Inflammation in Young Women with Self-Reported Rosacea: An Exploratory Cross-Sectional Study

Supplementary Table S1

Three-group sensitivity analysis. Values are medians. Kruskal–Wallis (KW) with post-hoc pairwise Mann–Whitney U. R = Rosacea (n = 19), H = Healthy Controls (n = 91), C = Comorbid Controls (n = 190). Microbiota values are relative abundances (%). Not FDR-corrected.

Host-side parameters

Variable	Rosacea	Healthy Ctrl	Comorbid Ctrl	KW p	R vs H	R vs C
BMI (kg/m²)	23.837	21.878	22.315	0.055	0.012	0.077
Body weight (kg)	67.000	62.000	63.500	0.024	0.006	0.061
Sleep Quality Score	32.000	27.000	29.000	0.013	0.006	0.098
WHO-5 Well-being	15.000	16.000	15.000	0.102	0.128	0.646
Erythrocytes ($\times 10^{12}$/L)	4.600	4.530	4.565	0.034	0.025	0.082
Monocytes ($\times 10^{9}$/L)	0.570	0.460	0.500	0.058	0.015	0.056
MCHC (g/L)	328.000	332.000	332.000	0.043	0.012	0.066
HOMA-B (%)	141.100	115.800	123.550	0.021	0.008	0.024
Calprotectin (mg/L)	1.020	0.660	0.810	0.054	0.026	0.150
Cortisol (nmol/L)	382.800	355.400	344.050	0.157	0.115	0.050
Shannon Entropy	7.198	7.662	7.640	0.227	0.070	0.125

Microbiota and functional modules

Feature	Rosacea	Healthy Ctrl	Comorbid Ctrl	KW p	R vs H	R vs C
Bifidobacterium	0.571	1.863	1.983	0.027	0.015	0.007
Roseburia	1.405	2.998	3.431	0.067	0.093	0.024
Parasutterella	0.066	0.107	0.150	0.055	0.073	0.020
Prevotellaceae NK3B31	0.111	0.078	0.060	0.037	0.117	0.015
Akkermansia	0.350	0.094	0.064	0.047	0.272	0.057
Vit. B12 production module	32.800	27.310	27.150	0.127	0.044	0.063
Gut barrier function module	18.230	14.080	15.615	0.061	0.042	0.294
Inflammation module	28.085	24.630	26.665	0.127	0.134	0.559

Supplementary Table S2

PICRUSt2 MetaCyc pathway analysis. All pathways enriched in rosacea. No pathway survived FDR correction (m = 394; lowest q = 0.768). PICRUSt2 pathways are inferred from 16S taxonomic abundances and are not independent of compositional findings.

Pathway	MetaCyc ID	p-value	Cluster
TCA cycle VIII (Helicobacter)	REDCITCYC	0.030	Energy
D-galactarate degradation I	GALACTARDEG-PWY	0.034	Carbohydrate
L-histidine degradation II	PWY-5028	0.037	Amino acid
Adenosylcobalamin salvage from cobinamide II	PWY-6269	0.044	B12 cluster
Adenosylcobalamin biosynthesis I	PWY-5509	0.045	B12 cluster
Adenosylcobalamin salvage from cobinamide I	COBALSYN-PWY	0.045	B12 cluster
L-arabinose degradation IV	PWY-7295	0.048	Carbohydrate
L-tryptophan degradation to 2-amino-3-carboxymuconate semialdehyde	PWY-5651	0.049	Trp/Kyn
NAD biosynthesis II from tryptophan	NADSYN-PWY	0.049	Trp/Kyn
Lysine fermentation to acetate and butanoate	—	0.094	Trend
Heparin degradation	—	0.100	Trend
Fucose degradation	—	0.100	Trend

Supplementary Methods

Classifier Specifications

An exploratory Random Forest classifier was trained using scikit-learn (v1.3) with the following hyperparameters: 500 trees, max_depth = 3, min_samples_leaf = 3, class_weight = ‘balanced’, random_state = 42. Two feature sets were evaluated: (1) microbiota-only: five genera (*Bifidobacterium*, *CAG-56*, *Anaerostignum*, *Roseburia*, *Comamonas*); (2) integrated: the same five genera plus HOMA-B and cortisol.

Feature selection was performed on the full dataset prior to cross-validation, which introduces optimistic bias. Nested cross-validation was not feasible given n = 18 rosacea cases. Leave-one-out cross-validation (LOOCV) served as the primary performance estimate. Five-fold stratified CV (10 repeats) was computed as a secondary estimate.

As exploratory validation, LOOCV-predicted rosacea probabilities were correlated (Spearman) with serum calprotectin—a monocyte-derived inflammatory marker not included in training (n = 187). Participants were dichotomized at median predicted probability; high- vs low-risk calprotectin levels were compared by Mann–Whitney U.

Supplementary Figure S1

Exploratory integrated microbiota–metabolic classifier (hypothesis-generating). (A) Leave-one-out cross-validated (LOOCV) ROC curves for the candidate models, including the microbiota-only and the integrated microbiota-plus-clinical Random Forest classifiers; the dashed grey diagonal denotes the line of no discrimination (AUC = 0.5), shown as a reference. (B) Correlation between LOOCV-predicted rosacea probability and serum calprotectin, an inflammatory biomarker not used in model training (*n* = 187); the dashed line is the ordinary least-squares regression fit through the individual data points, shown to visualise the direction of the association reported by the Spearman correlation in the inset. (C) Random Forest feature importance (Gini), with bars colored by feature domain (microbiota versus clinical). All performance estimates are cross-validated; the classifier is exploratory and is not proposed as a diagnostic tool.

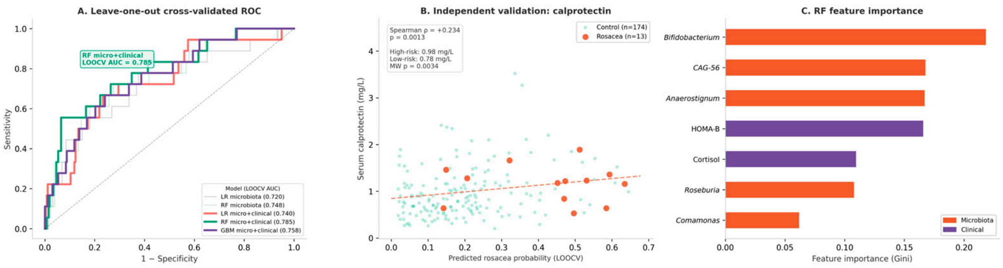

Supplementary Results

PICRUSt2 Functional Pathway Analysis

Among the 15 Biomes-derived functional modules, only the vitamin B12 production module was nominally significant (32.8 vs 27.2; $p = 0.048$). PICRUSt2 analysis of 394 testable MetaCyc pathways identified 10 nominally significant pathways (all enriched in rosacea) and 18 trend-level pathways ($p < 0.10$). Three belonged to the cobalamin biosynthesis cluster: adenosylcobalamin salvage from cobinamide II (PWY-6269; $p = 0.044$), adenosylcobalamin biosynthesis from cobyrinate a,c-diamide I (PWY-5509; $p = 0.045$), and adenosylcobalamin salvage from cobinamide I (COBALSYN-PWY; $p = 0.045$).

Two tryptophan-related pathways were enriched: L-tryptophan degradation to 2-amino-3-carboxymuconate semialdehyde (PWY-5651; $p = 0.049$) and NAD biosynthesis II from tryptophan (NADSYN-PWY; $p = 0.049$). Both feed into the kynurenine pathway. Additional significant pathways included TCA cycle VIII ($p = 0.030$), D-galactarate degradation I ($p = 0.034$), L-histidine degradation II ($p = 0.037$), and L-arabinose degradation IV ($p = 0.048$). No pathway survived per-level FDR correction (lowest $q = 0.768$).

Exploratory Correlation Analyses (Full Cohort, n = 283)

The gut barrier function module was negatively correlated with BMI ($\rho = -0.117$, $p = 0.050$), and the vitamin B12 production module with BMI ($\rho = -0.142$, $p = 0.017$). Shannon diversity showed a trend-level negative correlation with HOMA-IR ($\rho = -0.109$, $p = 0.070$).

Within the rosacea subgroup ($n = 18$), Shannon Entropy was negatively correlated with the inflammation indicators module ($\rho = -0.523$, $p = 0.026$) and trended with HOMA-IR ($\rho = -0.424$, $p = 0.079$). The predicted vitamin B12 production module did not correlate with serum vitamin B12 in the full cohort ($\rho = 0.066$, $p = 0.265$) or rosacea subgroup ($\rho = 0.059$, $p = 0.817$), suggesting the elevated module reflects a compositional shift rather than a direct effect on host B12 status.

Supplementary Figure S2

Spearman correlation matrix (full cohort, n = 283). Microbiota taxa (red), functional modules (green), systemic markers (blue). Values = Spearman ρ. *p < 0.05, **p < 0.01, ***p < 0.001.

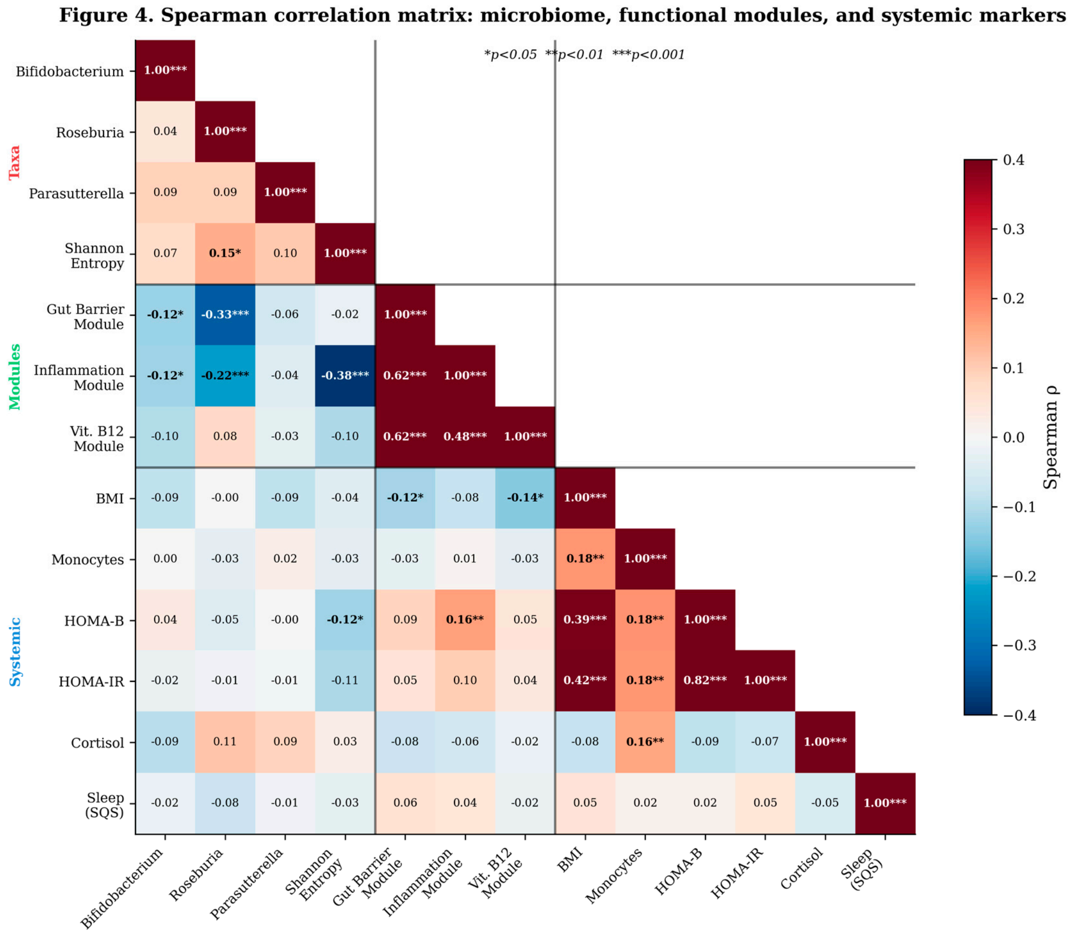

Supplementary Checklist S1: STROBE Statement for Cross-Sectional Studies

von Elm E, et al. Strengthening the Reporting of Observational Studies in Epidemiology (STROBE) statement: guidelines for reporting observational studies. BMJ. 2007; 335(7624):806–808.

Item	Topic	Recommendation	Reported in
1	Title and abstract	(a) Indicate the study's design with a commonly used term in the title or abstract (b) Provide an informative and balanced summary	Title page ("Exploratory Cross-Sectional Study"); Abstract (Background, Methods, Results, Conclusions)
2	Background/rationale	Explain the scientific background and rationale for the investigation	Section 1 (Introduction), paragraphs 1–4
3	Objectives	State specific objectives, including any prespecified hypotheses	Section 1, final paragraph; Section 2.5 (pre-specified sensitivity analysis)
4	Study design	Present key elements of study design early in the paper	Section 2.1 (cross-sectional analysis within cohort)
5	Setting	Describe the setting, locations, and relevant dates	Section 2.1 (Zagreb, Croatia); 2.3 (Gut Microbiome Center, University Hospital Sveti Duh)
6	Participants	(a) Give eligibility criteria, sources and methods of selection (b) For matched studies, give matching criteria and number of exposed and unexposed	Section 2.1 (inclusion/exclusion criteria; N=300, rosacea n=19, controls n=281)
7	Variables	Clearly define all outcomes, exposures, predictors, potential confounders, and effect modifiers	Sections 2.2–2.4 (questionnaires, anthropometrics, blood markers, 16S sequencing); 2.5 (BMI adjustment, sensitivity analysis)
8	Data sources/measurement	For each variable of interest, give sources of data and details of methods of assessment (measurement). Describe comparability of assessment methods if there is more than one group	Sections 2.2 (validated questionnaires: WHO-5, PSS, SQS, MDD-W, MEDAS); 2.3 (standardized anthropometry, fasting blood); 2.4 (16S V3–V4 sequencing, QIIME2, SILVA v138.1)
9	Bias	Describe any efforts to address potential sources of bias	Section 2.1 (exclusion criteria: antibiotics <3 mo, pregnancy, infections); 2.5 (BMI adjustment, three-group sensitivity); 4.4 (Limitations: self-report, unmeasured confounders listed)
10	Study size	Explain how the study size was arrived at	Section 2.1 (N=300 from parent cohort; n=19 rosacea by self-report); 4.4 (power limitations discussed)
11	Quantitative variables	Explain how quantitative variables were handled in the analyses	Section 2.5 (median [IQR]; Mann–Whitney U; per-level BH FDR; rank-based BMI residuals; HOMA indices calculated)
12	Statistical methods	(a) Describe all statistical methods (b) Describe any methods to examine subgroups/interactions (c) Explain how missing data were addressed (d) If applicable, describe analytical methods for follow-up (e) Describe any sensitivity analyses	Section 2.5 (Mann–Whitney U, PERMANOVA, per-level FDR, global FDR, BMI adjustment, three-group Kruskal–Wallis, Random Forest classifier, LOOCV, calprotectin correlation); Supplementary Methods
13	Participants	(a) Report numbers of individuals at each stage of study (b) Give reasons for non-participation at each stage (c) Consider use of a flow diagram	Section 3.1 (N=300; microbiota n=283; rosacea n=19/18)
14	Descriptive data	(a) Give characteristics of study participants (b) Indicate number of participants with missing data for each variable	Tables 1–2; Section 3.1 (comorbidity prevalence); 2.3 (subset sizes for extended panels: n=94–200)
15	Outcome data	Report numbers of outcome events or summary measures	Sections 3.1–3.3 (all medians, IQRs, p-values reported); Tables 1–3

16	Main results	(a) Give unadjusted estimates and, if applicable, confounder-adjusted estimates and their precision (b) Report category boundaries when continuous variables were categorized (c) Consider translating estimates into meaningful clinical context	Sections 3.2–3.3 (unadjusted); 3.3 (BMI-adjusted); 3.4 (three-group); 3.5 (classifier AUC with CI)
17	Other analyses	Report other analyses done—e.g., analyses of subgroups and interactions, and sensitivity analyses	Section 3.4 (three-group sensitivity); 3.5 (classifier); Supplementary Results (PICRUSt2, correlations)
18	Key results	Summarise key results with reference to study objectives	Section 4, opening paragraph; 4.5 (Conclusions)
19	Limitations	Discuss limitations of the study, taking into account sources of potential bias or imprecision	Section 4.4 (Limitations: self-report, sample size, unmeasured confounders including PPIs, probiotics, stool consistency, hormonal status, dermatologic treatment)
20	Interpretation	Give a cautious overall interpretation considering objectives, limitations, multiplicity of analyses, and results from similar studies	Sections 4.1–4.3 (contextualised with 9 prior studies); 4.5 (explicitly framed as hypothesis-generating)
21	Generalisability	Discuss the generalisability (external validity) of the study results	Section 4.4, final paragraph (all-female, narrow age, European population)
22	Funding	Give the source of funding and the role of the funders	Funding section (EU NextGenerationEU, NPOO.C3.2.R3-I1.04.0073; funders had no role in study design or analysis)

Supplementary Checklist S2: STORMS Checklist for Human Microbiome Studies

Mirzayi C, et al. Reporting guidelines for human microbiome research: the STORMS checklist. Nat Med. 2021;27(11):1885–1892.

Section	Item	Reported in
Study design	1. Study type and design	Section 2.1 (cross-sectional within cohort); Title
Study design	2. Research objectives and hypotheses	Section 1, final paragraph; Section 2.5 (pre-specified sensitivity)
Participants	3. Setting and enrollment	Section 2.1 (Zagreb, Croatia; Jan 2024–Apr 2025)
Participants	4. Inclusion/exclusion criteria	Section 2.1 (age 30–35, female; excluded: antibiotics <3 mo, pregnancy, acute infection, oncology)
Participants	5. Participant characteristics and metadata	Tables 1–2; Section 2.2 (demographics, diet, lifestyle, comorbidities)
Laboratory methods	6. Sample collection, transport, and storage	Section 2.4 (self-collected stool, scientific.pro kit, stored −20°C)
Laboratory methods	7. DNA extraction method and kit	Section 2.4 (QIAamp 96 PowerFecal QIAcube HT Kit, Qiagen; Hamilton/Tecan automation)
Laboratory methods	8. Primer pair and variable region	Section 2.4 (V3–V4; modified Illumina 16S Metagenomic protocol)
Laboratory methods	9. Sequencing platform and chemistry	Section 2.4 (Illumina NextSeq 2000)
Computational methods	10. Quality filtering and chimera removal	Section 2.4 (min 150 bp, Phred ≥20; vsearch uchime2_ref v2.7.0 against SILVA v138.1)
Computational methods	11. Sequence clustering / denoising method	Section 2.4 (CD-HIT at 97% similarity)
Computational methods	12. Taxonomic classification database and version	Section 2.4 (SILVA v138.1; Yarza et al. 2014 thresholds)
Computational methods	13. Alpha and beta diversity methods	Section 2.5 (Shannon, Chao1, Pielou, Faith's PD via QIIME2/scikit-bio; Bray–Curtis PERMANOVA, 9999 permutations)
Computational methods	14. Normalization / transformation	Section 2.4 (relative abundances); 2.5 (rank-based residuals for BMI adjustment)
Computational methods	15. Differential abundance testing	Section 2.5 (Mann–Whitney U; per-level BH FDR; global FDR in Table S1)
Computational methods	16. Multiple testing correction	Section 2.5 (per-level BH FDR primary; global FDR sensitivity; binomial test of overall signal)
Computational methods	17. Software and versions	Section 2.5 (Python 3, scipy, pandas, scikit-learn, QIIME2, scikit-bio v0.5.6, PICRUSt2)
Results	18. Participant flow and final sample size	Section 3.1 (N=300 enrolled; microbiota n=283; rosacea n=19/18)
Results	19. Sequencing depth and quality	Section 2.4 (min 50,000 assigned reads per sample)
Results	20. Alpha and beta diversity results	Section 3.3 (Shannon p=0.093; PERMANOVA F=1.33, p=0.147)
Results	21. Differential abundance results	Section 3.3; Table 3 (15 nominally significant genera; lowest FDR q=0.165)
Results	22. Potential confounders addressed	Sections 3.3 (BMI adjustment); 3.4 (three-group sensitivity); 4.4 (unmeasured confounders listed)
Discussion	23. Reproducibility and generalisability	Section 4.1 (comparison with 9 prior studies); 4.4 (generalisability limitations)
Discussion	24. Study limitations	Section 4.4 (self-report, power, confounders, 16S resolution, PICRUSt2 limitations)
Data access	25. Data and code availability	Data Availability Statement (available from corresponding author; patent pending)
